# Joint Pain and Arthritis as First Clinical Manifestation of Systemic Amyloidosis and Multiple Myeloma: Case Report and Brief Literature Review

**DOI:** 10.3390/hematolrep14010004

**Published:** 2022-03-02

**Authors:** Francesco Mazziotta, Gabriele Buda, Maria Livia Del Giudice, Enrico Orciuolo, Edoardo Benedetti, Matilde Masini, Vincenzo De Tata, Sara Galimberti, Mario Petrini

**Affiliations:** 1 Hematology Division, Pisa University Hospital, 56126 Pisa, Italy; francemazziotta@gmail.com (F.M.); ga.buda@libero.it (G.B.); e.orciuolo@alumni.sssup.it (E.O.); edobenedetti@gmail.com (E.B.); sara.galimberti@unipi.it (S.G.); mario.petrini@unipi.it (M.P.); 2 Department of Translational Research and New Technologies in Medicine and Surgery, Pisa University Hospital, 56126 Pisa, Italy; matilde.masini@unipi.it (M.M.); vincenzo.detata@unipi.it (V.D.T.)

**Keywords:** immunoglobulin light-chain amyloidosis, multiple myeloma, plasma cell neoplasm, joint diseases

## Abstract

Amyloidosis is a rare disease that is often seen in conjunction with multiple myeloma (MM). Its damage varies depending on the anatomical site affected; however, it is believed that many cases of amyloidosis are misrecognized due to the fact that its signs and symptoms are nonspecific. Joint amyloidosis, in particular, may be confused with degenerative or autoimmune diseases. When it is associated with MM, it can significantly precede the diagnosis of the latter. We describe a case report of a woman of Nigerian heritage diagnosed with MM with widespread joint manifestations compatible with a diagnosis of amyloidosis, which had preceded the diagnosis of MM and benefited from MM treatment. Faced with the suspicion of amyloidosis, if confirmed, this can be used to anticipate the diagnosis of MM, and at a more advanced stage, it can benefit from the treatment of the MM.

## 1. Introduction

Amyloid light-chain (AL) amyloidosis is a rare plasma-cell dyscrasia caused by tissue deposition of amyloidogenic light chains in the form of abnormal, insoluble fibers [1,2,3].

Approximately 15% of patients affected by multiple myeloma (MM) may develop overt AL amyloidosis [1], but it is estimated that nearly 30% of MM patients have subclinical amyloid deposits in the fat pad, bone marrow, and other sites [1].

Symptoms of amyloidosis depend on the involvement of different organs. The signs and symptoms suggestive but not pathognomonic are tongue swelling, diarrhea, nausea and loss of appetite, fatigue, unexplained weight loss, shortness of breath, swelling of legs, and peripheral nerve damage (e.g., carpal tunnel syndrome, prickling, tingling, and numbness in the extremities) [1,2,3].

All these clinical conditions, combined with the alteration of peculiar biomarkers (NT-proBNP [4] and microalbuminuria), lead to systemic amyloidosis being suspected in the case of monoclonal gammopathy of undetermined significance and abnormal free light chain (FLC) ratio [1].

To confirm the diagnosis, a biopsy of affected organs, a bone marrow biopsy, or a subcutaneous fatty tissue aspirate must be performed [1,3]. Amyloidogenic proteins produce a characteristic apple-green birefringence after Congo red staining [5]. Electron microscopy can reveal the presence of amyloid protein, which appears as rigid aggregates of fibrils [6,7]; however, this technique is not still ordinarily recommended as a diagnostic tool.

## 2. Discussion

Since 2012, a woman of Nigerian heritage, then 57 years old, was followed at Hematology Division, Pisa University Hospital, for monoclonal gammopathy of uncertain significance (MGUS) with clinical evaluation and biannual laboratory tests.

The patient’s personal pathological history did not show clinically relevant aspects, except for several long-standing joint deformities, often painful, with perilesional edema and stiffness in movements and involvement of large and small joints, mainly of the right hand, with associated ulnar deviation and prehension deficit. Autoimmunity serum markers appeared negative.

In February 2015, the patient showed an increase in known joint pain and, although in the context of the relative stability of laboratory tests with suspicion of a hematological genesis of the disorders, she was re-evaluated with blood count and blood chemistry tests, as well as with histological and instrumental examinations.

**Figure 1:**
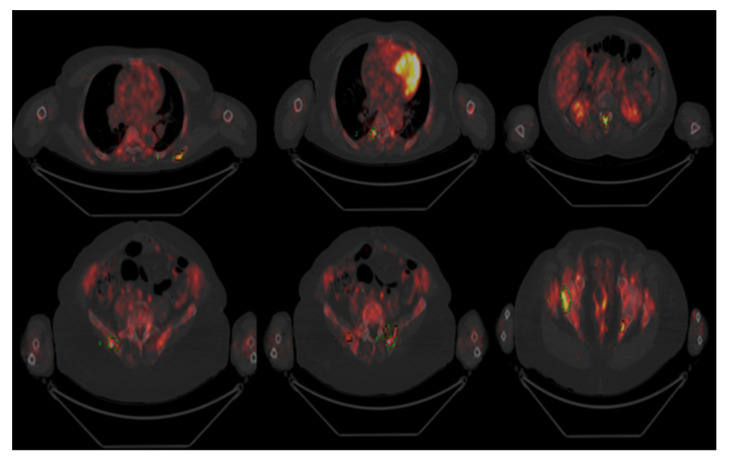
PET/CT scan: uptake in the right scapula, L1 vertebral level, and multifocal uptakes in the pelvis.

Bone marrow showed 80% infiltration of CD138-positive plasma cells, while PET/CT (Figure 1) revealed focal uptake of 18F-fluorodeoxyglucose at several sites: right scapula, with a maximum standardized uptake value (SUV) of 6.4; right eighth rib (SUV 6.1); L1 vertebral level (SUV 7.3); multifocal uptake in the pelvis (SUV 9.3).

**Figure 2:**
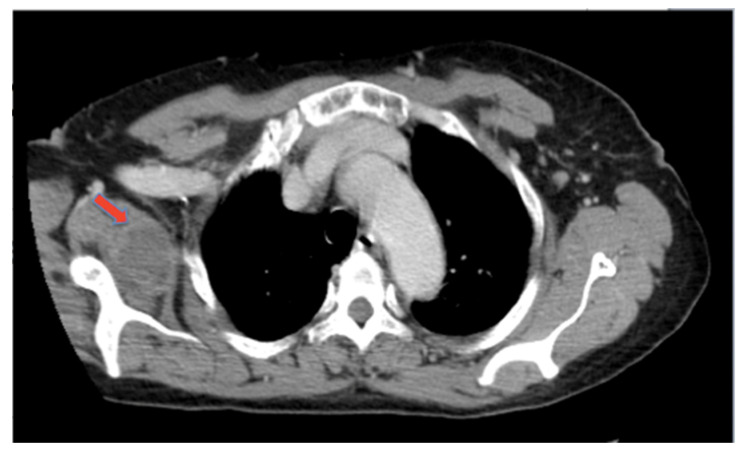
CT scan: density alteration of the right scapula and infiltrative involvement of subscapularis muscle.

A CT scan (Figure 2) also showed infiltrative involvement of the subscapularis muscle by a homogeneous hypo-dense tissue (PET negative).

Based on these investigations, a diagnosis of multiple myeloma (ISS stage 2) was made. However, due to the peculiar clinical features, further instrumental and histological examinations were performed.

The radiography of the right hand revealed signs of arthrosis involving the distal interphalangeal joints of the second and third finger and the proximal interphalangeal joint of the second finger.

**Figure 3:**
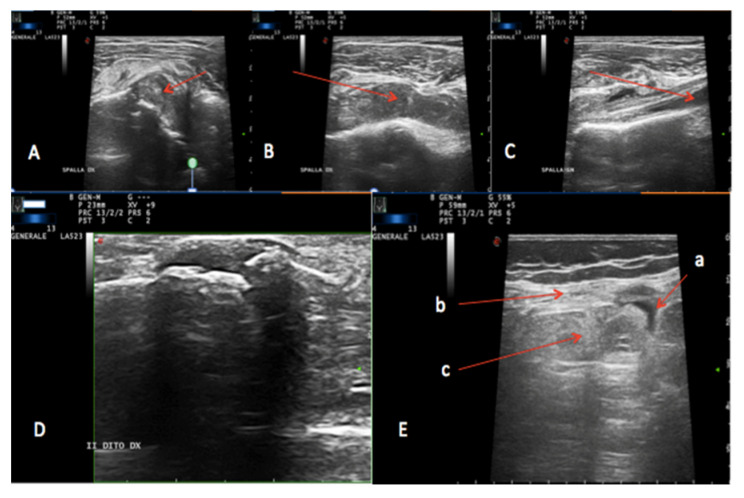
Ultrasound scan. (**A**–**C**): Left (**A**,**B**) and right (**C**) shoulder: hypoechoic solid material involving the acromioclavicular joint; (**D**) right hand second finger: hypoechoic solid material involving the proximal and middle phalange articulation; (**E**) right knee articulation: a. suprapatellar bursa, b. extensor tendon, c. dishomogeneous solid material below the extensor tendon material involving the acromioclavicular joint.

An ultrasound scan was performed at the same anatomical site, and the investigation was extended to both shoulders and to the right knee, which showed the presence of hypoechogenic solid material involving the examined joints (Figure 3).

**Figure 4:**
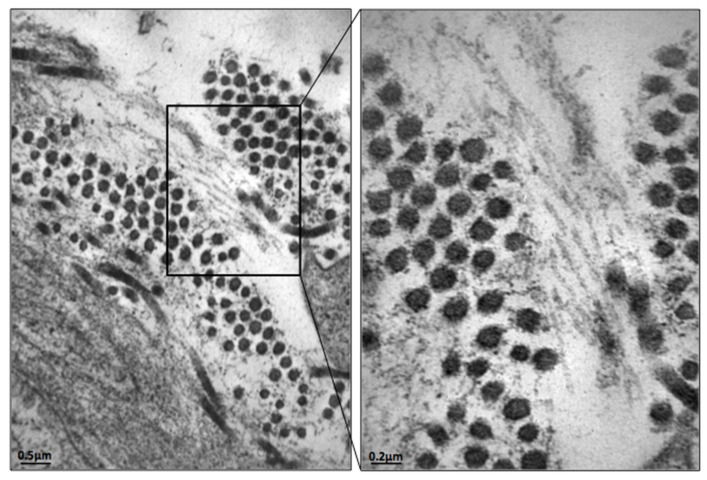
Periumbilical fat pad aspirate: electron microscopy image, positive for amyloid fibrils.

Even in the presence of negative echocardiography for wall thickening, there was a well-founded suspicion of an amyloid deposit: periumbilical fat aspirate confirmed this hypothesis by electron microscopy (Figure 4).

With the clinical priority to proceed to the treatment of MM and thanks to the evidence collected until that moment, the joint biopsy was postponed, and the patient was rapidly started on systemic chemotherapy.

The patient underwent induction therapy with four cycles of the combination of bortezomib, thalidomide, and dexamethasone (VTD) [7] at the end of which a partial response was recorded. With the prospect of autologous stem cell transplantation (ASCT), a successful autologous stem cell harvest procedure was attempted in December 2015.

The patient therefore continued the therapeutic course with an admission to our hematology unit and underwent a conditioning procedure with the administration of Melphalan 140 mg/m^2^; on 19 January 2016, she performed ASCT without complications.

Immediately subsequent follow-ups showed a significant reduction in arthropathy, an improvement in quality of life, and a resumption of normal relationship life, with a complete disease response achieved.

The last evaluation performed in April 2018 showed that the patient was still alive and with the persistence of the reported complete hematologic response.

## 3. Conclusions

Amyloidogenic light-chain deposition associated with MM may lead to an arthropathy resembling rheumatoid arthritis (RA) [8].

Amyloid arthropathy (AA) is a rare manifestation generally characterized by symmetric rheumatoid facto--negative nonerosive polyarthritis [8].

A literature review made by Ahmed M. Elsaman et al. [8] found only 101 reported patients with amyloid arthropathy between 1931 and 2012.

Its incidence in the population is unknown due to its rarity and lack of defining criteria. AA affects males and females with a ratio of 1:1 and generally occurs in older adults, with a median age of 60 years at diagnosis [8].

About 1/3 of patients are misdiagnosed as RA [8], even though the two conditions are pathogenetically and clinically different [8,9].

AA is a nonerosive polyarthritis, rarely monoarticular or oligoarticular, especially involving the shoulder joint, in the absence of rheumatoid nodules. The rheumatoid factor is negative, and patients often present joint involvement before the diagnosis of MM.

Unlike RA, AA is a chronic synovitis related to the phagocytosis of fibrillar amyloid by macrophages; the activation of the macrophage leads to the activation of pre-(IL)-1β to IL-1β and, as a consequence, to the activation of pro-inflammatory cascades in other cells. Thus, AA seems to be mediated by an undue activation of the innate immune system rather than by a lymphocyte-mediated mechanism [9].

Acquired light-chain amyloidosis in patients affected by MM is often a diagnosis of exclusion. Early diagnosis is a great challenge to physicians [1] because symptoms can be occult and mimic other disorders.

In our case, clinical suspicion of amyloid joint deposition was subsequently confirmed ex juvantibus by the resolution of arthropathy after the transplant.

Considering that AA often anticipates the diagnosis of MM [8], it is important to detect symptoms in its early stage (e.g., MGUS). Prompt recognition and specific treatment of this disorder represents a critical point to prevent MM complications and worsening of amyloid deposition.
